# Multidisciplinary Intervention and Acceptance and Commitment Therapy for Return-to-Work and Increased Employability among Patients with Mental Illness and/or Chronic Pain: A Randomized Controlled Trial

**DOI:** 10.3390/ijerph15112424

**Published:** 2018-10-31

**Authors:** Erik Berglund, Ingrid Anderzén, Åsa Andersén, Lars Carlsson, Catharina Gustavsson, Thorne Wallman, Per Lytsy

**Affiliations:** 1 Department of Public Health and Caring Sciences, Uppsala University, Box 564, SE-75122 Uppsala, Sweden; Ingrid.Anderzen@pubcare.uu.se (I.A.); Asa.Andersen@pubcare.uu.se (Å.A.); Lars.Carlsson@ltdalarna.se (L.C.); Catharina.Gustavsson@ltdalarna.se (C.G.); Thorne.Wallman@dll.se (T.W.); Per.Lytsy@pubcare.uu.se (P.L.); 2 Centre for Clinical Research Dalarna, Uppsala University, SE-79182 Falun, Sweden; 3 Centre for Clinical Research Sörmland, Uppsala University, SE-63188 Eskilstuna, Sweden; 4 Department of Clinical Neuroscience, Division of Insurance Medicine, Karolinska Institute, SE-17177 Stockholm, Sweden

**Keywords:** return-to-work, vocational rehabilitation, multidisciplinary rehabilitation, chronic pain, mental illness, sick leave

## Abstract

Background: People on long-term sick leave often have a long-lasting process back to work, where the individuals may be in multiple and recurrent states; i.e., receiving different social security benefits or working, and over time they may shift between these states. The purpose of this study was to evaluate the effects of two vocational rehabilitation programs, compared to a control, on return-to-work (RTW) or increased employability in patients on long-term sick leave due to mental illness and/or chronic pain. Methods: In this randomized controlled study, 427 women and men were allocated to either (1) multidisciplinary team management, i.e., multidisciplinary assessments and individual rehabilitation management, (2) acceptance and commitment therapy (ACT), or (3) control. A positive outcome was defined as RTW or increased employability. The outcome was considered negative if the (part-time) wage was reduced or ceased, or if there was an indication of decreased employability. The outcome was measured one year after entry in the project and analyzed using binary and multinomial logistic regressions. Results: Participants in the multidisciplinary team group reported having RTW odds ratio (OR) 3.31 (95% CI 1.39–7.87) compared to the control group in adjusted models. Participants in the ACT group reported having increased employability OR 3.22 (95% CI 1.13–9.15) compared to the control group in adjusted models. Conclusions: This study of vocational rehabilitation in mainly female patients on long-term sick leave due to mental illness and/or chronic pain suggests that multidisciplinary team assessments and individually adapted rehabilitation interventions increased RTW and employability. Solely receiving the ACT intervention also increased employability.

## 1. Introduction

Common mental illness and chronic pain are the two most frequent reasons for long-term sick leave in many countries, including Sweden [1,2]. In addition to the individual suffering related to mental illness and chronic pain, there is a major public/societal burden related to productivity losses [3]. In 2008/2009, the social insurance system in Sweden was reformed and maximum time sick-leave reimbursements were introduced; people on sick leave were transferred to the Swedish Public Employment Service (SPES) to have their work ability assessed [4]. During 2010–2012, about 40,000 people reached the maximum time for sick-leave compensation, and were among the first to have their work ability assessed by SPES, and thus be available for work in the labor market.

People on long-term sick leave often have a long-lasting process back to work [5], where the individuals may be in multiple and recurrent states; i.e., receiving different social security benefits or working, and over time they may shift between these states. This includes shifts between different social security benefits, programs, and part-time and full-time work [5]. Even if individuals do not necessarily move directly to return-to-work (RTW), they might come closer to, or further apart from, the labor market. Longer periods of sick leave are also known as a risk factor for not RTW [6,7]. Other factors that are associated with less RTW are female gender, age, pain, disability, depression, high work demands, previous sick leave, unemployment, and activity limitations [8].

Several strategies have been proposed to prevent work absence and facilitate RTW, and these interventions can be broadly classified as unimodal (for example individual psychotherapy), multimodal programs (team-based assessments and synchronized treatment by several health professionals), and interventions that target a structural level [9]. A recent systematic review of workplace interventions for RTW for musculoskeletal, pain-related, and mental health conditions concluded that there is strong evidence that multimodal intervention encompassing at least two of the three domains shortens duration away from work [10]. The current intervention project, which was carried out between 2010–2012, targeted people on long-term sick leave and at risk of losing their reimbursement from social insurance. Previous studies have foremost targeted people having short-term or medium-term sickness absence [10]. It is still not fully explored how people on very long-term sick leave RTW after experiencing mental illness or chronic pain. The aim of the study was to investigate the effects on RTW or changes in employability for people on long-term sick leave. The objective was to analyze the effects of two vocational rehabilitation interventions: a multidisciplinary team assessment and individualized treatment and/or unimodal psychotherapy with ACT intervention in patients on sick leave due to common mental disorders or chronic pain. To study an empirically relevant intervention outcome that reflects a stepwise rehabilitation perspective, individuals shifting between sources of income was used as an indicator of increased or decreased employability and RTW.

## 2. Materials and Methods 

This study was conducted as a randomized controlled trial (RCT) and was implemented in two phases. In the first phase, only female participants were allocated to the multidisciplinary treatment (MDT) intervention group, the acceptance and commitment therapy (ACT) intervention group, or to the control group. In the second phase, both women and men participants were allocated either to the MDT group or the control group. The study sample from phase 1 has being used in previous studies [11,12]. The present study evaluates pooled data from both phases.

### 2.1. Subsection

**Figure 1:**
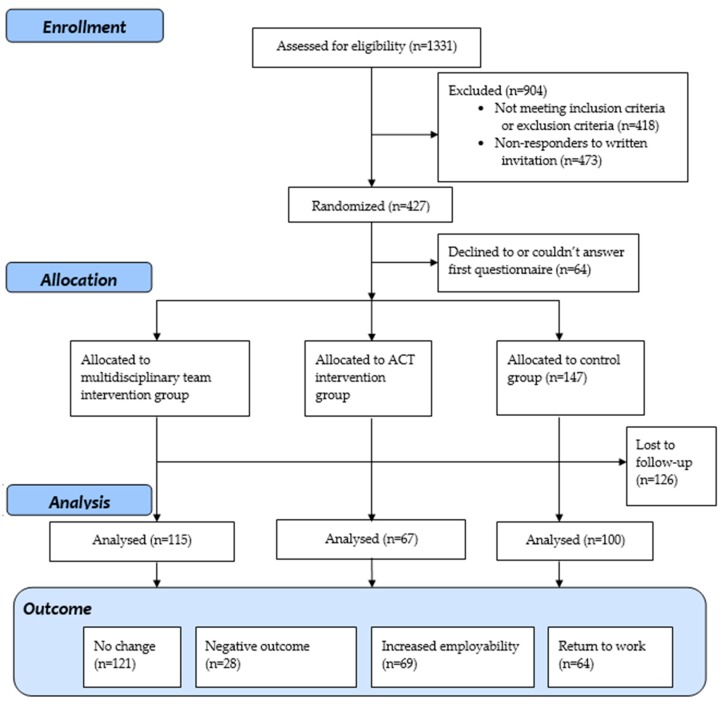
Flow chart of inclusion and follow-up procedure.

Participants eligible for the study were women and men (men only in phase 2) on long-term sick leave or a temporary disability pension due to a mental illness and/or pain-related diagnosis in Uppsala County, Sweden. Mental illnesses included F-diagnoses (with the exceptions of the exclusion criteria diagnosis), and pain diagnoses included M-diagnoses and R-diagnoses defined in the International Statistical Classification of Diseases and Related Health Problems 10th revision (ICD-10). The office of the Swedish Social Insurance Agency (SSIA) identified 1331 individuals on sick leave, with these problems expected to reach their maximum time of sick leave between 2010–2012. After first inclusion, the individuals’ sickness certificates were screened by a physician and an occupational therapist or psychologist to determine fulfilment of the inclusion criteria, and to ensure that they did not fulfil the exclusion criteria. The inclusion criteria were: (1) on sick leave for mental illness and/or chronic pain; (2) aged between 20–64 years. The exclusion criteria were: (1) at high risk for suicide; (2) ongoing alcohol/substance abuse; (3) major mental illness (schizophrenia, bipolar disorder type I, severe social dysfunction/personality disorder); (4) participation in psychotherapy or another vocational rehabilitation program. The SSIA initially identified 1331 people as eligible for the project; after screening, 418 were excluded from the project due to not meeting the inclusion criteria or meeting the exclusion criteria. The remaining 913 individuals were then contacted by mail with information about the project and invited to participate. Out of these, 473 did not respond or declined to participate. A further 13 people were excluded from the research part of the study (but received care in accordance with their allocation) due to being contacted before the formal approval by the ethics committee. The remaining 427 gave their consent to participate, and were randomized by the SSIA into the multidisciplinary team group (n = 178), ACT group (n = 102), or control group (n = 147). Out of these, 282 participants answered the outcome measure (see Figure 1). The inclusion, randomization, and allocation were performed consecutively during the project, about three to four months ahead of the date to match when participants were expected to transition to the SPES. Initially, the participants had an equal chance of being allocated into either the MDT, ACT, or control groups. In phase 2 of the study, two-thirds were randomized to the MDT group and one-third was allocated to the control group. The second phase also included men; the reason for also including men was an increased need among men for this type of intervention.

### 2.2. Interventions 

The interventions started one to three months ahead of each participant’s expected transfer to the SPES. Patients randomized to the MDT group met individually with professionals from a multidisciplinary team, including a psychologist (PS), a physician (MD), an occupational therapist (OT), and a social worker (SW). The health professional assessed the participant’s strengths and limitations for RTW from each perspective. The team then met without the participant to establish an individualized rehabilitation plan, which was later brought back to the participant by one of the team members. The participants had the choice of accepting all, none, or parts of the rehabilitation plan’s suggestions and interventions. The plan could involve further examinations, assessments, meetings, or psychotherapy (which then was delivered by the PS) for up to a period of one year. The team met weekly during the project period to evaluate the situation and synchronize the planned or ongoing activities for each participant. Participants allocated to the ACT group received solely ACT treatment. ACT is psychological therapy that uses cognitive behavioral therapy (CBT) and acceptance and mindfulness strategies, together with behavioral strategies, to increase function and quality of life rather than decreasing symptoms to increase psychological flexibility, which has shown to be of value for a number of long-lasting conditions [13]. Most psychotherapy sessions and meetings, for both the multidisciplinary team and ACT group, took place at the clinic, but there was also a possibility of conducting meetings at the participant’s home, work, or elsewhere. Sessions were typically about an hour long.

In the second phase of the project, the possibility of being randomized to sole ACT treatment (ACT group) was omitted. The reason was that during phase 1, it turned out that some participants randomized to the ACT group were not interested in receiving ACT, but were interested in receiving other types of treatments. Participants randomized to the MDT group (in both phases) had the option of receiving ACT if suggested by the psychologist after assessment. In addition to the treatments, all of the participants also had scheduled collaboration meetings with their administrator at the SPES, the SSIA, and their contact person within the project. These meetings aimed to ascertain the rehabilitation plan and its goal for the individual as well as for the participating organizations.

The control group did not receive any intervention organized within the intervention project, but was free to receive the usual assistance and care provided by their regular contact with the SSIA, SPES, and potential contacts with healthcare providers. The control group responded to the same follow-up questionnaires as the intervention groups.

### 2.3. Questionnaires and Outcome Measures

Data collected before the intervention included demographics, such as the respondent’s gender, age, and educational level (categorized as compulsory school, secondary school or equivalent, or university). Factors related to mental health were assessed using the Hospital Anxiety and Depression Scale (HADS) [14]. The HADS is a 14-item scale; seven of the items relate to anxiety, and seven relate to depression. The General Self-Efficacy scale (GSE) was used to measure the participants’ perceived self-efficacy [15]. The GSE consists of 10 statements and is answered on a four-point Likert scale ranging from 1 = “Not at all true” to 4 = “Completely true”. The items were summed to give a total score of 10 to 40. A mean value was calculated as the sum of all of the answers divided by the number of statements, as long as no more than three statements were missing [16]. The current version of the GSE has been translated into Swedish and has been validated [17]. There is no definite cut-off score for the self-efficacy scale. In this study, the mean value (2.3) of self-efficacy was used to categorize participants with lower (<2.3) versus higher (≥2.3) self-efficacy. The work-related predictor variables that were used in this study included employment contract status (unemployed or employed), extent of sick leave (full-time or part-time), and years with income replacement, which were collected through SSIA registry data.

To measure intervention outcomes, the participants responded to a mailed follow-up questionnaire 12 months from randomization (see Figure 1).

This study’s primary outcome uses self-reported sources of income at baseline and follow-up at 12 months to create an outcome variable with four exclusive categories that were believed to capture both changes in RTW as well as changes in employability. Outcome was measured by using the following question: “How do you provide for yourself?” Participants indicated answers in percentages of income from various sources currently and a year earlier. Income sources included: wages (through employment or own business); sickness compensation from the SSIA; and compensation through the SPES. The answers were then compared. If a person reported having increased their proportion of wage due to more work as source of income at 12 months, then they were categorized as “RTW” (having returned-to-work in full or to some degree). A person was categorized as having an “increased employability” if the compensation change indicated increased availability for work, such as increased compensation from the SPES instead of sick leave compensation from the SSIA, as an indication that the person was (more) eligible for work in the labor market. Outcome was considered “negative” if the (part-time) wage was reduced or stopped, or if there was an indication of decreased employability (increased compensation from the SSIA without increased wage). An unchanged outcome was defined as having no changes in wage, employability, or sickness benefits. The outcome variable was further dichotomized for the binary logistic regression analysis into those having a positive (having RTW or increased employability) or negative (having negative or unchanged outcome) outcome.

### 2.4. Analyses

Differences in outcome between the two intervention groups and the control group were investigated using Chi-square tests. The intervention’s effects were analyzed using binary and multinomial logistic regression models. In randomized controlled trials, confounders are believed to be equally distributed between compared groups. However, it might be of interest to investigate the associations among different factors to the outcome. It might be of further interest to adjust for confounders that are potentially associated with the attrition. To adjust for potential predictors and confounders and other variables that may be associated with the outcome, a stepwise approach was performed using sets of variables. In model 1, the intervention group was adjusted for age and education level. In model 2, additional adjustments were made for HADS, self-efficacy, employment contract, extent of sick leave, and years with income replacement.

Multinomial logistic regressions were used comparing a negative outcome, increased employability, and RTW to those with no changes as a reference category. Multinomial logistic regressions were also performed and adjusted for potential confounders. A complete case analysis was performed in which respondents answered the outcome measure; also, due to a large number of missing values in the outcome variable (34.0%), a sensitivity analysis was performed, in which missing participants were assumed to have made no change in outcome (reference category). All of the tests were two-sided, and a level of *p* < 0.05 was considered statistically significant. The statistical analyses were performed using SPSS statistics (IBM Corp, Armonk, NY, USA), version 22.0.

### 2.5. Ethical Considerations and Trial Registration

All of the participants provided written informed consent for the study. The first phase of the study was approved by the Regional Ethical Review Board of Uppsala in 2010 (Reg. no. 2010/088) and the extension (second phase) of the project was approved in 2011 (Reg. no. 2010/088/1). In the reporting of the results of this trial, we have tried to follow the Consolidated Standards of Reporting Trials (CONSORT) checklist as far as possible. The study was registered at the Clinicaltrials.gov Register Platform (ID NCT03343457) on 15 November 2017 (retrospectively registered).

## 3. Results

The average age of the study group was 48.9 years (SD 8.3). The study group consisted of 94.7% women and 5.3% men. Secondary school was the most common completed education level. Most of the participants (68.1%) had an employer, and the average time on sick leave was 7.7 years (SD 3.2). About half of the participants were on full-time sick leave, and half were on part-time sick leave.

One-third of the participants were on sick leave for psychiatric disorders, about one-third were on sick leave for pain-related conditions, and about one-third were on sick leave for a combination of psychiatric and pain-related disorders. Common pain-related problems were fibromyalgia and pain in the back, neck, or joints. Common mental disorders were depression (current and recurrent) and stress-related and anxiety disorders.

Participants in the multidisciplinary team group attended on average 4.4 (SD 5.4) sessions delivered by the MD, OT, PT and SW, and 4.7 (SD 6.4) sessions with a psychologist who provided ACT, for a total average of 9.1 (SD 8.4) sessions. Participants in the ACT group attended on average 8.0 (SD 6.0) sessions with a psychologist providing ACT, as shown in Table 1. Of the complete cases in this study, 66% of the participants in the MDT group received at least one meeting with a psychologist providing ACT as one part of their individual plan.

### 3.1. Return-to-Work, Increased Employability, Negative Outcome, or No Change

At the 12-month follow-up, 64 participants (22.7%) had RTW, 69 participants (24.5%) had increased employability, 28 participants (9.9%) had a negative outcome, and 121 participants (42.9%) had not changed in any direction. Among participants in the multidisciplinary team, 31.1% had RTW, 27.0% had increased employability, and 4.3% had a negative outcome. Among participants in the ACT group, 17.9% had RTW, 35.8% had increased employability, and 9.0% had a negative outcome, as shown in Table 2. 

### 3.2. Logistic Regression Models

The adjusted binary logistic regression analysis showed that the MDT group had an OR of 4.62 (95% CI 2.27–9.41) for RTW or increased employability. The corresponding number for the ACT group was OR 2.35 (95% CI 1.07–5.19), as shown in Table 3.

### 3.3. Multinomial Regression Models

The adjusted multinomial logistic regression analysis showed that the multidisciplinary team group had an odds ratio (OR) of 3.31 (95% CI 1.39–7.87) for RTW, an OR of 4.24 (95% CI 1.60–11.26) for increased employability, and an OR of 0.19 (95% CI 0.05–0.72) on negative outcome. The adjusted multinomial logistic regression analysis showed that the ACT intervention group had an OR of 3.22 (95% CI 1.13–9.15) on increased employability, but no significant effect on RTW. See Table 4.

Due to a large number of internal missing values in the outcome measure (34.0%), a multinomial logistic regression analysis was also performed, in which non-responding participants were assumed to have made no change (reference category) in outcome. Similar results, despite somewhat lowered effects, showed that the multidisciplinary team group had an adjusted OR of 2.57 (95% CI 1.19–5.58) on RTW; an adjusted OR of 2.80 (95% CI 1.20–6.52) on increased employability; and an adjusted OR of 0.18 (95% CI 0.05–0.62) on negative outcome. The ACT intervention group had an adjusted OR of 2.79 (95% CI 1.12–6.97) on increased employability.

## 4. Discussion

This study aimed to investigate the effects on RTW and increased employability of two vocational rehabilitation interventions in patients on long-term sick leave due to mental illness and/or chronic pain. In this study, a non-dichotomous outcome was used to capture not only RTW, but also changes in increased or decreased employability. The results indicate that multidisciplinary interventions and individualized treatments may increase RTW and employability in patients on long-term sick leave due to common mental illness and/or chronic pain. The results in this study are similar to results presented in other studies regarding RTW [18,19,20,21,22,23]. Also, in review studies, this type of multi-domain intervention seems to be the most effective intervention for RTW outcome when managing musculoskeletal and pain-related conditions [10]. The results also suggest that multidisciplinary team and ACT interventions may increase employability, i.e., transferring individuals in the insurance system to increased availability for employment and work. 

One reason for removing the separate ACT group in the second phase of the project was that the intervention providers discovered that some people who were randomized to ACT were not keen on receiving psychological therapy [24]; this might also explain the somewhat weaker outcome of the ACT group.

At the time when the study was conducted, the participants were about to lose their sickness insurance benefits due to a major change in the social insurance system. These circumstances may have led to the inclusion of participants who would not otherwise have been motivated to participate in vocational rehabilitation, thus creating a study population with relatively low motivation and belief in their own ability to RTW. Since longer periods of sick leave and absence from the labor market are known risk factors for RTW failure [6,7], this project was believed to target a difficult group. Taking all of the factors into account, including the interventions, the multinomial regression explained 37.8% (r^2^) of the outcome, which leaves a 62.2% unexplained variance that is dependent on factors that were not assessed in the model. This indicates that there are other factors outside the intervention program that affect the outcomes. For instance, this study did not include any work-directed modifications or interventions, which is seen as a factor to increase the chances of RTW goals [25]. The causal chain between sick leave, mental illness, and chronic pain is not fully explored. Sick leave is warranted by an inability to work, but sick leave may in itself also contribute to depression, unhealthy living, and stress [26]. Previous research has identified several important factors for RTW [8]. Current demands in the labor market are important for re-entry to the workforce, including factors on a systemic/organizational level [27]. Many of these factors are not affected by health and rehabilitation interventions such as this project. Instead, several of these factors are found “upstream” in the analogy of a “river”, which is used to describe how previous social, financial, environmental, and historic factors ultimately go on to profoundly influence present outcomes [28]. In the present study, this implies that even if a participant in the project reduces their ill health and increases employability, these results might not be captured in a sole RTW outcome; thus, RTW is (also) dependent on upstream factors that are determined outside the reach of the project. Especially when considering the long-term sick leave period among the project participants (on average 7.7 years), it is reasonable to assume that the labor market has changed in several aspects during their absence, such as work content and demands etc. Therefore, an outcome measure was constructed that also captures changes in different transition stages made by program participants, such as increased employability. This does not apply to all of the participants; several had part-time jobs. 

In Sweden, as well as in many other countries, concern and efforts provided by society for people on sick leave are regulated by different laws and principals and divided among different authorities. There have been doubts about whether a multi-actor model is optimal, and limitations have been found in the system. This project included several authorities and professions with one collaborative goal: to support the participants in their vocational rehabilitation and increase their likelihood for RTW. This project was also a collaborative challenge, as different professions in the MDT worked closely with one goal. It should be noted that the MDT and ACT groups received about the same total number of sessions in the project. The ACT group only received sessions with an ACT psychologist; the MDT group received on average about half of the sessions from an ACT psychologist and half from the other professions. 

The major findings in this study imply that a multidisciplinary team-based intervention directed at people on long-term sick leave, including ACT counseling, seems to help people, even those on long-term sick leave, to RTW. The findings add to the evidence that multidisciplinary interventions such as vocational rehabilitation may increase RTW among patients with mental illness and/or chronic pain [29].

There is a need to further investigate multidisciplinary RTW interventions, determine their core components, and consider combining them with workplace collaboration, including the employer and interventions in the work environment.

### Strengths and Limitations

The strengths of this study include the randomized prospective controlled design and the experimental design, which suggest that the effects on RTW are in fact effects of the interventions.

This study also has some limitations. Reliable evidence for RTW should be assessed using information on validated employment and work activity. In this study, the outcome was based on self-reported data and actual RTW was not measured; instead, a changed proportion of income source was used as an indicator for changes in working status and employability. However, similar studies often use proxy variables for measuring RTW [23]. Since the data—both exposures and outcomes—are self-reported, there is a risk of recall bias. Another limitation of this study is that education level, HADS, and self-efficacy were measured after randomization, so the scores could have already been influenced by the knowledge of intervention group affiliation. The results were not adjusted by type of disease (mental illness or chronic pain). 

Many participants did not show up, dropped out, or did not answer the follow-up questionnaires, which weakens the assessment of the outcomes. There could be different reasons for the high attrition: perhaps the participants did not actually want to be part of the project, were too sick to participate, or felt they had a problem that would not benefit from the project.

Since participants in the treatment groups received individual treatment as well as organizational collaboration, this raises the question of which of these interventions mediated the effect, if either. The reason to include organizational collaboration was to set and mutually agree on each individual’s RTW goal. Another potential problem with this study is that the two types of multidisciplinary team interventions that were combined were not identical, but they were similar.

## 5. Conclusions and Practice Implications

This study of vocational rehabilitation in men and women on long-term sick leave due to mental illness and/or chronic pain showed that multidisciplinary team assessments and individually adapted rehabilitation interventions increased RTW and employability. Sole ACT intervention increased employability.

The study implies that it is possible to increase RTW among people on long-term sick leave due to mental illness and/or chronic pain with a multidisciplinary rehabilitation intervention. Multidisciplinary team rehabilitation and sole ACT also seem to be useful for increasing employability and gradually moving people in a stepwise positive direction, through the welfare system, and eventually toward RTW. 

## Figures and Tables

**Table 1:** Characteristics of study participants.

Variable	Group/Measure	MDT Group	ACT Group	Control Group	Total
Sex	Female	90.4	100.0	93.9	93.9
	Male	9.6	0.0	6.1	6.1
Age, years	Mean (SD)	49.9 (8.5)	47.8 (7.8)	48.0 (8.3)	48.7 (8.3)
Education	Compulsory school	22.2	14.7	21.1	20.1
	Secondary school or equal	48.9	44.0	46.5	46.9
	University	28.9	41.3	32.5	33.0
HADS ^a^	Anxiety, mean (SD)	10.9 (5.0)	10.1 (4.9)	11.1 (5.3)	10.8 (5.1)
	Depression, mean (SD)	9.5 (4.7)	8.5 (4.2)	9.1 (5.1)	9.1 (4.7)
SE ^b^	SE, mean	2.3 (0.7)	2.4 (0.7)	2.3 (0.7)	2.3 (0.7)
Employment contract	Employed	66.3	57.8	61.9	62.8
	Not employed	33.7	42.2	38.1	37.2
Extent of sick leave	Full-time	55.7	53.9	55.2	55.1
	Part-time	44.3	46.1	44.8	44.9
Years with income replacement	Mean (SD)	8.1 (3.3)	7.6 (3.1)	7.5 (3.2)	7.8 (3.2)
Dosage of intervention	Sessions with psychologist, mean (SD)	4.7 (6.4)	8.0 (6.0)	0.0 (0.0)	3.9 (5.9)
	Sessions with MD ^c^. OT ^d^. PT ^e^ and SW ^f^, mean (SD)	4.4 (5.4)	0.0 (0.0)	0.0 (0.0)	1.8 (4.1)
	Total sessions in the project, mean (SD)	9.1 (8.4)	8.0 (6.0)	0.0 (0.0)	5.7 (7.4)

Figures as percentages if not stated otherwise. ^a^ Hospital Anxiety and Depression Scale, ^b^ Self-efficacy, ^c^ Physician, ^d^ Occupational therapist, ^e^ Physical therapist, ^f^ Social worker. ACT: acceptance and commitment therapy.

**Table 2:** Return to work, increased employability, negative, or unchanged outcome according to different groups.

Outcome	MDT Group	ACT Group	Control Group	Overall
RTW (%)	31.3 **	17.9 **	16.0 **	22.7
Increased employability (%)	27.0 **	35.8 **	14.0 **	24.5
Negative outcome (%)	4.3 **	9.0 **	17.0 **	9.9
No change (%)	37.4 **	37.3 **	53.0 **	42.9

Pearson Chi-Square test was used for proportions. ** *p* ≤ 0.01. RTW: return-to-work.

**Table 3:** Binary logistic regressions presenting odds ratios (OR) of reporting return to work or increased employability vs. negative or unchanged outcomes.

Variable	Group/Measure	Crude OR (95% CI)	Model 1 OR (95% CI)	Model 2 OR (95% CI)
Intervention group	Group			
	Control (ref.)	1	1	1
	MDT^a^ group	3.26 ** (1.85–5.74)	4.22 ** (2.18–8.15)	4.62 ** (2.27–9.41)
	ACT^b^ group	2.71 ** (1.42–5.16)	2.42 * (1.17–4.97)	2.35 * (1.07–5.19)
Demographic	Age	0.96 * (0.94–0.99)	0.96 * (0.92 - 0.99)	0.96 * (0.93–1.00)
	Education level			
	Compulsory school	1	1	1
	Secondary school-equal	1.87 (0.91–3.82)	1.60 (0.74–3.46)	1.98 (0.85–4.58)
	University	1.24 (0.58–2.66)	1.23 (0.55–2.77)	1.28 (0.53–3.11)
Health and work related factors	HADS^c^, Anxiety	0.99 (0.94–1.04)		1.05 (0.96–1.14)
	HADS^c^ Depression	0.97 (0.92–1.02)		0.97 (0.89–1.06)
	Self-efficacy (<2.30)	1		1
	Self-efficacy (≥2.30)	1.76 * (1.07–2.89)		2.75 ** (1.33–5.72)
	Employment contract			
	Not employed	1		1
	Employed	0.70 (0.42–1.15)		0.78 (0.38–1.61)
	Extent of sick leave			
	Full time	1		1
	Part time	0.67 (0.42–1.07)		0.56 (0.29–1.10)
	Years with income replacement	0.97 (0.90–1.05)		0.97 (0.88- 1.06)
Nagelkerke r^2^			14.0%	24.1%

Odds ratio (OR), 95% CI: 95% confidence interval. * *p* < 0.05, ** *p* < 0.01. ^a^Multidisciplinary treatment. ^b^Acceptance and Commitment Therapy. ^c^Hospital Anxiety and Depression Scale, ranging from 0 to 21. Model 1 = Intervention group + age + education level, Model 2 = Model 1 + HADS + Self-efficacy + Employment contract + Extent of sick leave + Years with income replacement.

**Table 4:** Results of multinomial logistic regression of intervention group’s effect on negative outcome, return to work, or increased employability.

Variable		Return to Work or Change in System Position					
		Negative Outcome		Increased Employability		Return to Work	
		Crude OR (95% CI)	Adjusted OR (95% CI)	Crude OR (95% CI)	Adjusted OR (95% CI)	Crude OR (95% CI)	Adjusted OR (95% CI)
Intervention group	Group						
	Control (ref.)	1	1	1	1	1	1
	MDT^a^ group	0.36 (0.12–1.06)	0.19 * (0.05–0.72)	2.73 ** (1.29–5.77)	4.24 ** (1.60–11.26)	2.77 ** (1.36–5.66)	3.31 ** (1.39–7.87)
	ACT^b^ group	0.75 (0.26–2.13)	0.36 (0.10–1.35)	3.63 ** (1.61–8.19)	3.22 * (1.13–9.15)	1.56 (0.66–3.86)	1.36 (0.48–3.86)
Demographic	Age	0.99 (0.94–1.04)	0.94 (0.88–1.01)	0.95 ** (0.92–0.99)	0.94 * (0.90–0.99)	0.97 (0.93–1.01)	0.96 (0.92–1.01)
	Education level						
	Compulsory school	1	1	1	1	1	1
	Secondary school or equal	1.41 (0.40–4.91)	0.76 (0.16–3.57)	3.78 * (1.31–10.92)	4.52 * (1.29–15.87)	1.16 (0.49–2.77)	1.08 (0.40–2.89)
	University	1.44 (0.40–5.22)	0.61 (0.13–2.98)	1.79 (0.57–5.64)	2.24 (0.58–8.71)	1.10 (0.45–2.74)	0.85 (0.30–2.39)
Health and work-related factors	HADS^c^, Anxiety	0.93 (0.85–1.01)	0.92 (0.80–1.06)	0.99 (0.93–1.06)	1.02 (0.92–1.14)	0.96 (0.90–1.02)	1.05 (0.94–1.16)
	HADS^c^, Depression	0.93 (0.85–1.02)	1.14 (0.96–1.35)	0.96 (0.90–1.02)	0.98 (0.87–1.10)	0.96 (0.89–1.02)	1.01 (0.90–1.13)
	Self-efficacy (<2.30)	1	1	1	1	1	1
	Self-efficacy (≥2.30)	1.99 (0.84–4.73)	1.81 (0.52–6.34)	1.74 (0.93–3.25)	2.71* (1.07–6.91)	2.35 * (1.22–4.50)	3.31 ** (1.34–8.15)
	Employment contract						
	Not employed	1	1	1	1	1	1
	Have work	3.96 * (1.13–13.93)	1.66 (0.32–8.70	0.52 * (0.28–0.95)	0.64 (0.27–1.54)	1.55 (0.78–3.11)	1.09 (0.44–2.71)
	Extent of sick leave						
	Full time	1	1	1	1	1	1
	Part time	15.65 ** (3.55–68.94)	34.57 ** (3.84–311.31)	0.56 (0.30–1.05)	0.44 (0.17–1.02)	1.65 (0.89–3.05)	1.42 (0.62–3.21)
	Years, with income replacement	1.07 (0.94–1.22)	1.07 (0.89–1.29)	0.99 (0.90–1.09)	0.99 (0.89–1.12)	0.98 (0.89–1.07)	0.96 (0.85–1.07)

Reference outcome category: No change. For the adjusted model, Nagelkerke r^2^ = 37.8%. * *p* < 0.05, ** *p* < 0.01. ^a^Multidisciplinary treatment. ^b^Acceptance and Commitment Therapy. ^c^Hospital Anxiety and Depression Scale, ranging from 0 to 21. Odds ratio (OR), significant level and confidence interval (CI) for having made RTW or (positive) system position change or negative change.
